# Mechanical Evaluation of Hydrogel–Elastomer Interfaces Generated through Thiol–Ene Coupling

**DOI:** 10.1021/acsapm.2c01878

**Published:** 2023-01-30

**Authors:** Khai D. Q. Nguyen, Stéphane Dejean, Benjamin Nottelet, Julien E. Gautrot

**Affiliations:** † Institute of Bioengineering, Queen Mary, University of London, Mile End Road, London E1 4NS, U.K.; ‡ School of Engineering and Materials Science, Queen Mary, University of London, Mile End Road, London E1 4NS, U.K.; § Polymers for Health and Biomaterials, IBMM, Univ Montpellier, CNRS, ENSCM, 34293 Montpellier, France

**Keywords:** hydrogel, elastomer, thiol−ene, norbornene, silicone, ε-caprolactone

## Abstract

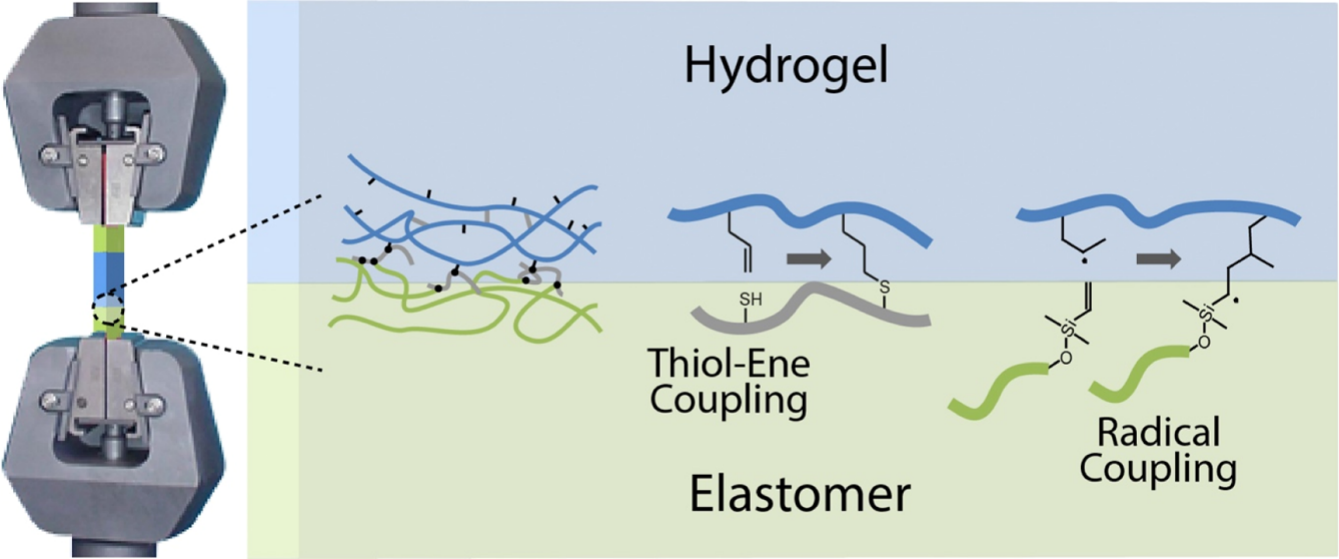

The formation of hybrid hydrogel–elastomer scaffolds is an attractive strategy for the formation of tissue engineering constructs and microfabricated platforms for advanced in vitro models. The emergence of thiol–ene coupling, in particular radical-based, for the engineering of cell-instructive hydrogels and the design of elastomers raises the possibility of mechanically integrating these structures without relying on the introduction of additional chemical moieties. However, the bonding of hydrogels (thiol–ene radical or more classic acrylate/methacrylate radical-based) to thiol–ene elastomers and alkene-functional elastomers has not been characterized in detail. In this study, we quantify the tensile mechanical properties of hybrid hydrogel samples formed of two elastomers bonded to a hydrogel material. We examine the impact of radical thiol–ene coupling on the crosslinking of both elastomers (silicone or polyesters) and hydrogels (based on thiol–ene crosslinking or diacrylate chemistry) and on the mechanics and failure behavior of the resulting hybrids. This study demonstrates the strong bonding of thiol–ene hydrogels to alkene-presenting elastomers with a range of chemistries, including silicones and polyesters. Overall, thiol–ene coupling appears as an attractive tool for the generation of strong, mechanically integrated, hybrid structures for a broad range of applications.

## Introduction

Hydrogels generated through thiol–ene coupling, by the reaction of thiol residues with alkenes, are increasingly used for the encapsulation of cells within 3D hydrogels and the design of soft materials for drug release.^1−3^ This enables us to achieve a broad control of physico-chemical properties that, in turn, can regulate the cell phenotype,^4−7^ the delivery of therapeutics,^8,9^ and the repair of soft tissues.^10−13^ Indeed, the excellent control of crosslinking through Michael addition and radical mechanisms, even in the presence of air and in physiological conditions, allows the control of mechanical properties of the resulting hydrogels while enabling the incorporation of a broad range of eukaryotic cells with excellent viabilities.^4,14−17^ In addition, the incorporation of protease cleavable moieties and physical supramolecular crosslinks allows us to confer cell-mediated degradability and viscoelastic properties to the resulting materials, essential to support gradual cell spreading and responsible to control the cell phenotype.^5,18^

Similarly, a number of thiol–ene-based elastomers have been developed, making use of the rapid crosslinking of the associated residues in air.^19−22^ Hence, an increasing number of reports proposed the crosslinking of polyurethanes, polycarbonates, and silicones using thiol–ene coupling and enabling their application in 3D printing platforms such as filament extrusion and stereolithography as well as for microfabrication.^19,20,23−28^ Taking advantage of the off-stoichiometry of thiol–ene-based resins also enables the control of bonding of microfluidic platforms^29^ and integration of microarrays, allowing the mechanical stimulation of cell cultures.^30^ In addition, thiol–ene and thiol–yne reactive polymers and elastomers allow us to confer biofunctionalization to traditional rigid degradable polymers such as poly(ε-caprolactone) (PCL) and poly(lactic acid). For example, propargylated PCL offers attractive functionality via thiol–yne coupling or click–azide reactions for the biofunctionalization of degradable implantable materials for tissue engineering applications.^31−33^

Increasingly, the potential of multiphase biomaterials combining relatively rigid hydrophobic elastomeric segments and softer hydrophilic hydrogels for a broad range of biomedical applications is emerging. This ranges from advanced in vitro models making use of hydrophobic elastomeric structures to compartmentalize and mechanically stimulate soft cell-encapsulating domains^34−38^ to the engineering of tissue engineering scaffolds.^39−41^ Such materials and associated microfabricated platforms are attractive for the study, control, and repair of soft–hard tissue interfaces.^42,43^ Although the engineering of a thiol–ene-based soft hydrogel enabling cell encapsulation, such as in gelatin methacrylate systems, has enabled their integration within a broad range of polyesters^44−47^ or silicones,^48,49^ the study of their mechanical integration and adhesion to corresponding elastomers has received relatively little attention. New generations of microfabricated and 3D printed microfluidic chips to be used as advanced in vitro models and tissue engineering scaffolds combining structural hydrophobic elastomers or polyesters and engineered cell-remodelable hydrogels will be attractive tools for bioengineers and scientists in the field of regenerative medicine. Strategies enabling us to mechanically integrate and provide strong bonding between such materials are therefore important.

In this study, we generate samples formed of two elastomer segments sandwiching a hydrogel phase that can be clamped for direct tensile testing and characterization of the mechanical properties and adhesion of the resulting hybrid samples. We examine the impact of radical thiol–ene coupling to crosslink both the silicone elastomer and hydrogels on the mechanics and failure behavior of the resulting hybrids. We first compare different types of hydrogels (two generated via thiol–ene coupling and one conventional acrylate gel) prior to the investigation of thiol–ene silicone in comparison with a commercial silicone, Sylgard. We examine, in particular, the toughness and failure behavior of these samples. Finally, we investigate the mechanics and failure properties of a propargylated polyester (PCL) bonded to a thiol–ene gel via direct coupling between thiol residues of the hydrogel matrix to the alkyne side chains of the modified PCL. This study demonstrates the strong bonding achieved by thiol–ene coupling and the associated toughness, in particular when bonding hydrogel and elastomeric phases both relying on this chemistry.

## Materials and Methods

### Materials and Equipment

Sylgard 184 polydimethylsiloxane (PDMS) was purchased from Dow Corning Corporation. Poly(ethylene glycol) diacrylate (PEGDA, *M*_n_ ∼ 575 g/mol), poly(ethylene glycol) dithiol (PEGDT, *M*_n_ ∼ 1000 g/mol), 2,2-dimethoxy-2-phenylacetophenone (DMPA or Irgacure 651), 2-hydroxy-4′-(2-hydroxyethoxy)-2-methylpropiophenone (Irgacure 2959, 98%), and dichloromethane (99.5%) were obtained from Sigma-Aldrich. Poly(mercaptopropylmethylsiloxane) (PDMS-SH) (*M*_n_ = 4000–7000 g/mol; mercaptopropyl siloxane unit content is 100%) and α,ω-vinyl-terminated PDMSs (vinyl-PDMS; *M*_w_ 28,000, 1000 cSt) were obtained from ABCR GmbH, Germany. Sodium carboxymethylcellulose (CMC, DP 1100, *M*_w_ 250,000, and degree of substitution, 0.7) was provided by GlaxoSmithKline UK. Phosphate buffer saline (PBS) solution was prepared by adding a tablet of PBS in 200 mL of deionized water. Photoirradiation was carried out using an Omnicure series 1500 lamp, and an ILT 1400-A radiometer photometer from International Light Technologies was used to measure the corresponding irradiation intensities.

### Synthesis of Propargylated Poly-ε-caprolactone (PCL-Alkyne)

Propargylated PCL was synthesized according to a protocol reported in the literature.^50^*M*_n_ 20,000 g/mol, PDI 2.1 (GPC). Propargylation level (^1^HNMR), 11%. Representative GPC and ^1^HNMR spectra obtained for this material can be found in the Supporting Information (Figures S1 and S2).

### Formation of PEGDA Hydrogel Precursor Solutions

PEGDA was dissolved in PBS (pH 7.4) at a concentration of 500 mg/mL. The stock solution of a photoinitiator (Irgacure 2959, 100 mg/mL in ethanol) and an additional volume of PBS were added to the polymerization mixture containing PEGDA stock to achieve final concentrations of the polymer of 125, 200, 300, and 400 mg/mL in PBS. The molar equivalent of the photoinitiator that was used was 1:40 with respect to PEGDA. The UV curable mixtures were finally vortexed thoroughly before use.

### Formation of Poly((2-(Methacryloyloxy)Ethyl) Dimethylpentenylammonium Chloride) (PDMAEMA-Pent) Thiol–Ene Hydrogel Precursor Solutions

PDMAEMA-Pent (degree of substitution, 99%) was synthesized according to a protocol reported in the literature.^51^*M*_n_ 68,000 g/mol (GPC). The full characterization is provided in our previous report.^51^ A stock solution of PDMAEMA-Pent was prepared with a concentration of 700 mg/mL in PBS (pH 7.4). To this PDMAEMA-Pent stock solution, PEGDT, 130 mg/mL in PBS, photoinitiator Irgacure 2959 (100 mg/mL in ethanol) and additional PBS were added to obtain the required concentration of PDMAEMA-Pent (200, 300, or 400 mg/mL). The molar equivalent ratio of alkene/thiol/initiator was kept at 1.2/1.0/0.1. The resulting photopolymerization mixture was further mixed by vortexing. Rheological characterization of PDMAEMA-pent hydrogels can be found in a previously reported literature.^51^

### Formation of Allyl Carboxymethylcellulose Hydrogel Precursor Solutions

Sodium CMC was functionalized with allyl groups using a protocol reported in the literature.^51^ The degree of allyl substitution was determined as 12% by ^1^HNMR. The full characterization is provided in our previous report.^51^ A stock solution of allyl-functionalized CMC (CMC-Allyl) was prepared in PBS at pH 6.0, and stock solutions of PEGDT and a photoinitiator (Irgacure 2959) were prepared in PBS and ethanol, respectively. To the CMC-Allyl solution, the PEGDT and Irgacure 2959 stock solutions were added to achieve a ratio of 1.2/1.0/0.1 between the allyl/thiol/initiator moieties. The mixtures were mixed by vortexing, followed by centrifugation to eliminate the bubbles formed. Rheological characterization of CMC-Allyl hydrogels can be found in a previously reported literature.^51^

### Preparation of UV Curable Silicone Samples (Thiol–Ene PDMS)

The UV curable resin was formulated from vinyl-PDMS of 1000 cSt, with a vinyl/thiol ratio of 1/2, with DMPA as the photoinitiator (0.1 equiv with respect to thiols). The resin was cast into a Teflon mold and irradiated with UV light (94 mW/cm^2^) for 2 min. The cured thiol–ene PDMS strips were cut into a size of 7 × 6 × 2 mm.

### Preparation of Sylgard 184 Silicone Samples

The base and curing agents were fully mixed at the weight ratio of 10:1 according to the manufacturer’s instructions. Bubbles in the mixture were removed by vacuum. The silicone mixture was then poured into a Teflon mold, followed by a curing reaction at 60 °C for 3 h. Once cured, the Sylgard strips were cut into 7 × 6 × 2 mm. In some samples, the cured Sylgard samples were coated with a mixture consisting of poly[(mercaptopropyl)methylsiloxane] (PDMS-thiol 100), a photoinitiator (Irgacure 651), and DCM. This solution was drop-cast onto a silicone surface while UV irradiation was applied through a quartz slide for 2 min. The treated surface was then washed with copious amounts of ethanol to remove any unreacted residue, followed by drying in a nitrogen flow.

### Preparation of Alkyne-Functionalized Poly-ε-Caprolactone (PCL-Alkyne) Samples

Alkyne-functionalized PCL powder was dissolved in chloroform, and the resulting solution was cast in Teflon molds, followed by drying in air overnight. The PCL-alkyne strips were finally cut into 7 × 6 × 2 mm.

### Bonding of Hydrogels to Elastomers to Formulate Hybrid Samples for Tensile Testing

The pre-cut silicones or PCL samples were painted 3 times with a solution containing the photoinitiator (Irg651 in DCM for silicone samples and Irgacure 651 in a mixture of DCM and ethanol (2:3) for PCL samples) using a cotton bud. Two elastomer strips were placed into Teflon molds, with a spacing of 5 mm. Hydrogel precursor solutions were poured into the gap defined by the two elastomer strips (Figure S3). The samples were then irradiated with UV light for 2 min at an intensity of 94 mW/cm^2^. Once cured, the resulting elastomer–hydrogel–elastomer hybrid structures were carefully removed. Finally, the samples were placed onto glass slides and kept hydrated until characterization.

### Tensile Mechanical Characterization

Tensile tests were carried out using an Instron 5566 frame at a tensile rate of 1.0 mm/min using a load cell of 5 N. Due to the low range of the load cell, a customized plastic clamp set was used. The hybrid samples were carefully clamped with a 1 mm elastomer material on either side of the hydrogel. Measurements were carried out using the software Bluehill 2 to control the Instron equipment, and data were acquired every 10 ms. A digital camera, Celestron, was used to monitor the failure behavior of the structure. The mechanical properties of hydrogels were determined from the stress–strain curve using the initial region of low extension (0–0.01 λ). For calculations of the moduli of the gels (apparent Young’s modulus), it was assumed that the elastomers used in our bonding experiments were not under significant strain, and thus, the length of the hydrogel segment (i.e., 5 mm) was considered as the length of the sample characterized. Although this assumption is incorrect, it afforded an apparent Young’s modulus that we could compare to the expected moduli of constitutive materials of the hybrids formed. The energy absorption per unit volume of material up to rupture, namely, tensile toughness, was determined from the integration of the stress–strain traces using Origin.

### Functionalization of Glass Substrates with Silanes

Glass slides were activated by plasma treatment using a plasma etcher (HPT-200, Henniker Plasma) before being immersed into silane solutions at room temperature overnight. The degree of thiol moieties on glass substrates was controlled by varying the molar ratios between mercaptopropyl trimethoxy silane and propyl trimethoxy silane in anhydrous toluene. The total concentration of silanes was fixed at 40 mM. The treated slides were rinsed thoroughly with toluene and isopropanol and kept away from dusts in a nitrogen box.

### Shear Lap Adhesion Testing

Glass substrates functionalized with different thiol densities were first placed facing each other at a distance of 1 mm. The photocurable PEGDA hydrogel solutions were then injected into the gap between two glass substrates until reaching the desired dimension (15 × 25 mm). The glass–gel–glass geometry was then irradiated by UV light (94 mW/cm^2^, 2 min) before being carefully placed into the tensile testing machine (Instron 3342 system with a 10 N static load cell and lap shear rig installed) for the shear lap experiments by tensile loading with a strain rate of 0.1 mm/mm/min. The shear adhesion strength was determined at the point of detachment, and the measurement was repeated in triplicate for each experimented condition.

### X-ray Photoelectron Spectroscopy

X-ray photoelectron spectroscopy (XPS) analysis was carried out using a Kratos Axis Ultra DLD electron spectrometer with an Al Kα X-ray source (1486.6 eV) operated at 150 W. A pass energy of 160 eV and a step size of 1 eV were used for survey spectra. For high-energy-resolution spectra, a pass energy of 20 eV and a step size of 0.1 eV were used. Sample charging effects were eliminated by correcting the observed spectra with the C 1s binding energy value of 285.0 eV.

### Cryo-Scanning Electron Microscopy

Cryo-scanning electron microscopy (Cryo-SEM) was carried out using a FEI Quanta 3D and a Gatan LTO2500 cryo-unit. The hybrid structure was cut and mounted onto the holder and flash frozen in slushy nitrogen. The sample was then transferred to the LTO2500 cryo-unit cooled to −140 °C and freeze-fractured using a cold scalpel to produce a freshly cut surface. The frozen water was sublimed off at −90 °C for 5 min, and the sample was sputter-coated with palladium. Afterward, the sample was transferred to the cryo-stage (−140 °C) for imaging.

## Results and Discussion

**Figure 1:**
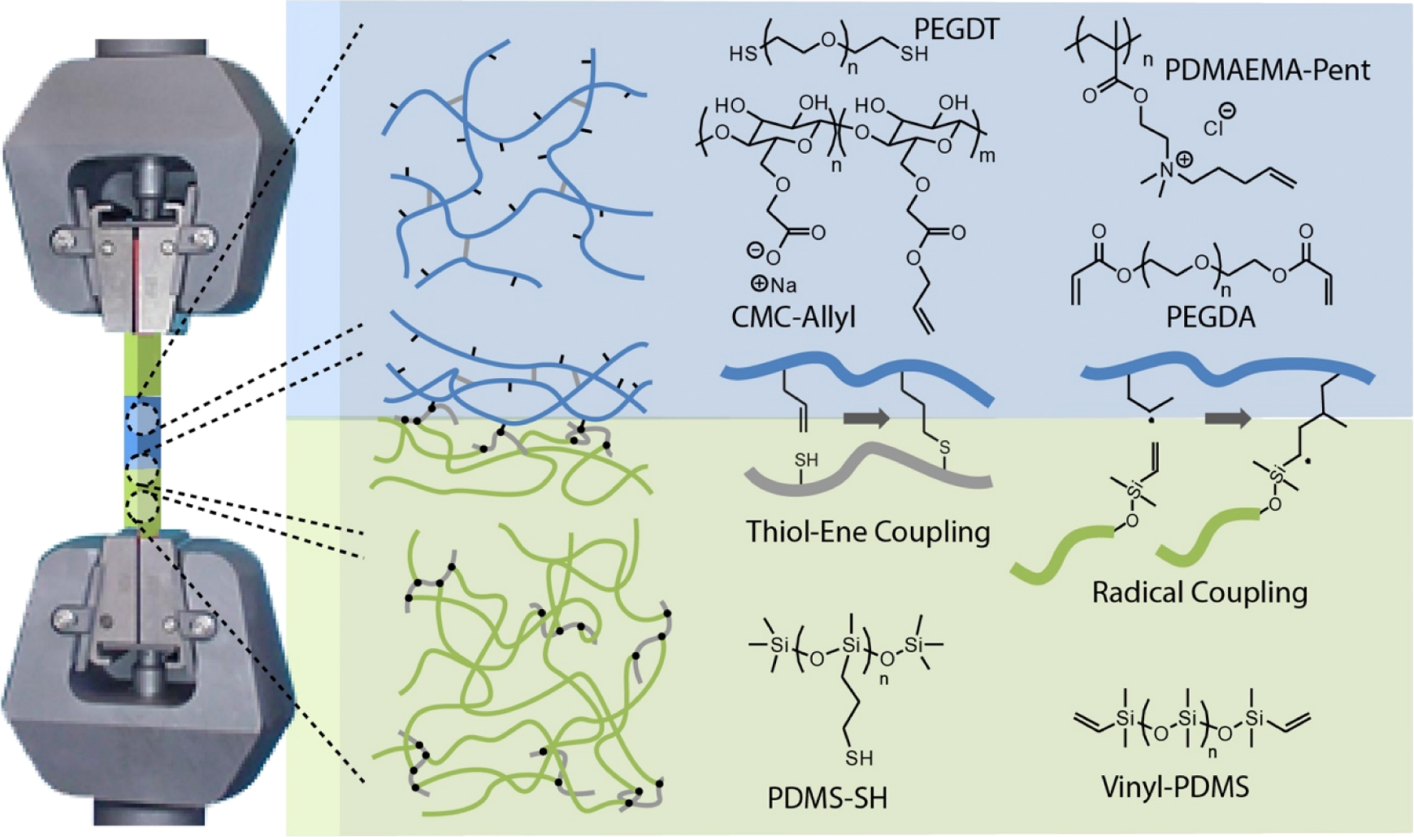
Schematic representation of the structure of the hybrid elastomer–hydrogel–elastomer samples formulated and their clamping for tensile testing. The chemical structure of the associated polymers and their expected bonding chemistry are presented.

To evaluate the mechanical properties of elastomer–hydrogel hybrids generated through thiol–ene coupling, we selected a thiol–ene PDMS formulation with a Young’s modulus of 0.25 MPa,^19^ formed through the combination of thiolated PDMS (PDMS-SH) and a vinyl-terminated silicone (vinyl-PDMS), together with a series of gels displaying a range of chemistries and mechanical properties (Figure 1). As a conventional acrylate-based gel, reactive through radical polymerization, PEGDA was selected, which can readily form hydrogels with relatively high moduli, depending on the monomer concentrations.^52,53^ In addition, two hydrogels cured through thiol–ene radical coupling were selected: polycationic hydrogels formed from PDMAEMA-Pent and polyanionic hydrogels formed from CMC functionalized with allyl residues (CMC-Allyl). These hydrogels can be readily crosslinked with poly(ethylene glycol) bis-thiol terminated (PEGDT) and afford materials with a moderate range of mechanical properties, depending on the polymer concentration and crosslinking ratio (ratio of thiol to alkene functions).^51^ PDMAEMA-Pent hydrogels, with their high degree of alkene functionality, displayed a broader range of mechanical properties (1–300 kPa). CMC-Allyl-based hydrogels displayed softer mechanical properties (0.5–20 kPa) as a result of their lower density of alkene residues, lower achievable concentration (higher viscosity), and a more moderate crosslinking efficiency.^51^ However, CMC-Allyl gels are relevant to the design of platforms, enabling the embedding and culture of cells in vitro, alongside other alkene-functionalized hydrogels formed through thiol–ene crosslinking from backbones such as hyaluronic acid, PEG, poly(2-alkyl-2-oxazoline), or dextran.^10,51,54,55^

**Figure 2:**
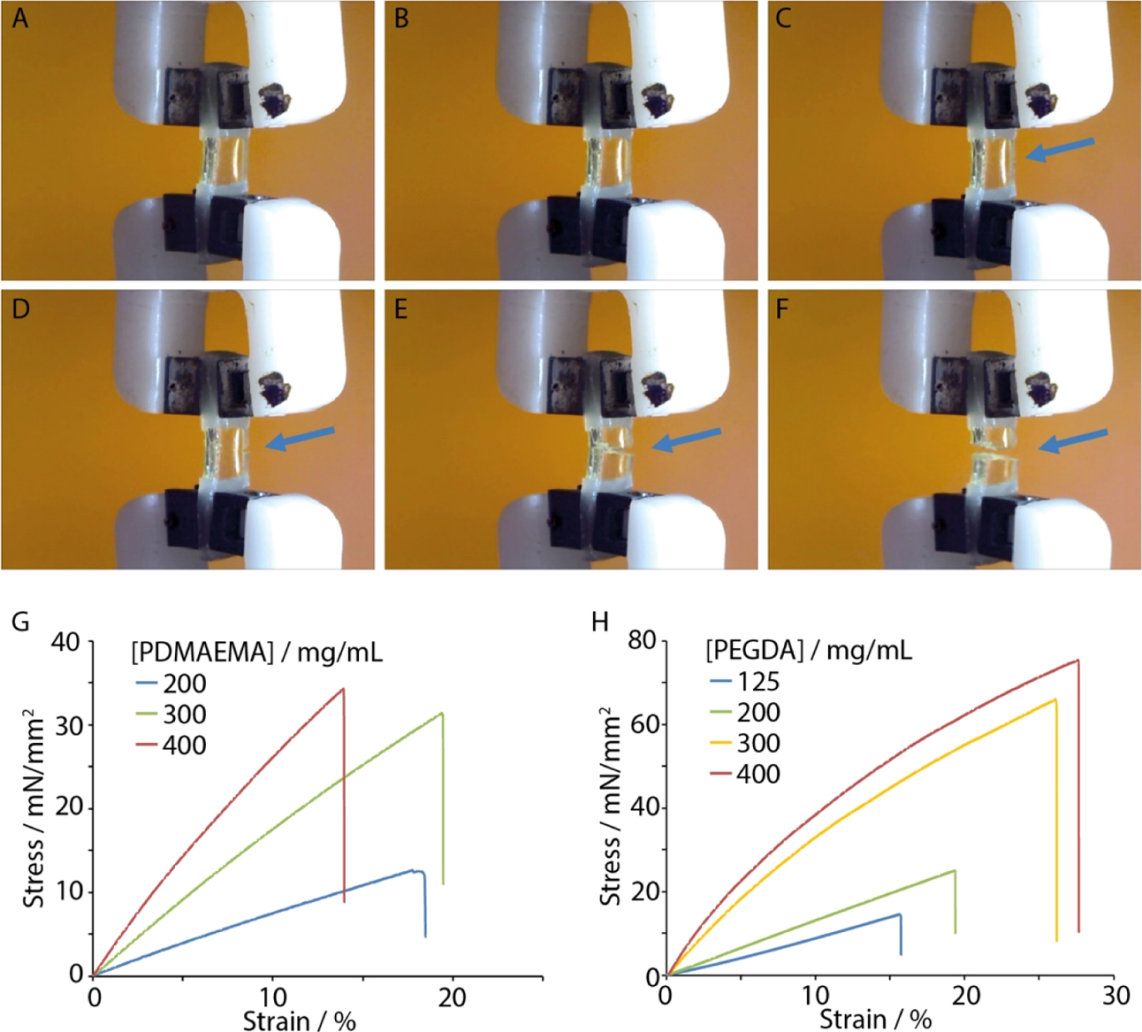
(A–F) Images of a hybrid sample (a 300 mg/mL PDMAEMA-Pent hydrogel bound to two thiol–ene PDMS strips) undergoing tensile testing (stretch rate, 1 mm/min). The blue arrows indicate the position at which sample failure occurs, demonstrating cohesive failure. (G,H) Representative examples of stress–strain traces recorded for hybrid samples formulated with thiol–ene PDMS and PDMAEMA-Pent (G) and PEGDA (H) hydrogels.

To prepare elastomer–hydrogel hybrids suitable for tensile testing, we generated elastomer samples in molds that were then cut and re-positioned to form 5 mm gaps in which hydrogel precursor solutions were deposited. After further photoirradiation to crosslink the hydrogel phase, the samples were carefully removed from the mold and mounted into the clamps of an Instron tensile tester. Therefore, the sample configuration generated allowed the direct mechanical testing of hybrids and probing of the bonding between different phases and the failure mechanism. In addition, this allowed the extraction of apparent Youngs’ moduli, mostly associated with the mechanical properties of the gel phase. Videos of the samples were captured during deformation to visualize sample deformation and investigate failure (Figure 2).

The deformation and mechanical properties of hybrids formulated from thiol–ene PDMS and thiol–ene hydrogel based on PDMAEMA-Pent were investigated first (Figure 2A–G). The samples tested displayed relatively linear tensile deformation profiles up to fracture, with relatively low elongations at break. The modulus clearly varied based on the initial polymer concentration used to formulate the gel, with a nearly fourfold increase when the polymer concentration doubled. In all cases, within the range of formulations tested, fracture of the hybrid samples was observed in the bulk of the hydrogel phase, indicating relatively strong adhesion sustained by thiol–ene bonding between the two phases. Similarly, hybrids generated between thiol–ene PDMS and PEGDA hydrogels displayed apparent moduli that increased gradually as a function of the monomer concentration in the range of 70–550 kPa over the range of formulations tested (Figure 2H).

**Figure 3:**
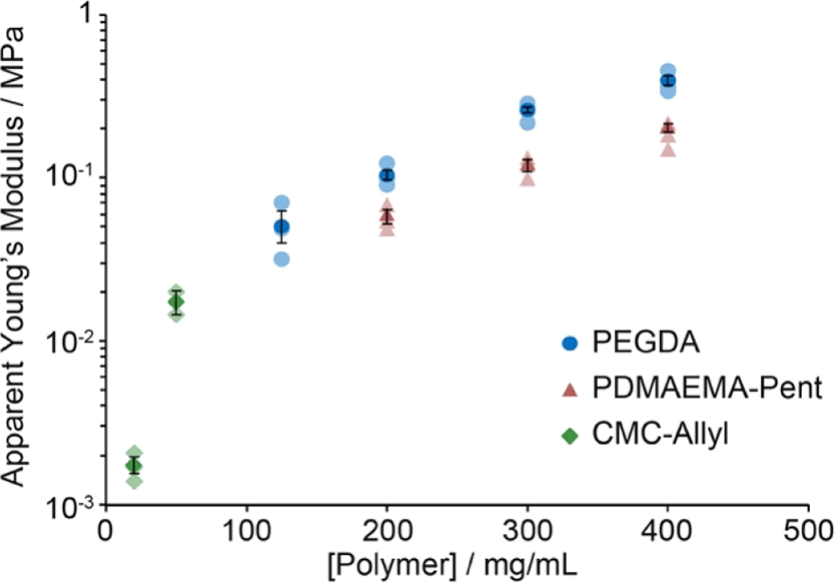
Apparent Young’s modulus of hybrid samples formulated with thiol–ene PDMS and PEGDA, PDMAEMA-Pent, and CMC-Allyl hydrogels at different polymer concentrations. Dark filled data points correspond to averages of ≥3 measurements ± standard errors. Light filled data points correspond to raw data points.

Hybrid samples generated from thiol–ene PDMS and CMC-Allyl hydrogels displayed significantly weaker mechanical properties (Figure S4). Not surprisingly, as relatively low polymer concentrations were used for the formulation of the hydrogel, the apparent Young’s modulus of the corresponding hybrids was significantly lower (2.4 and 24 kPa for hydrogels formulated from 20 and 50 mg/mL CMC-Allyl, respectively; Figure 3). However, CMC-Allyl-based hybrids displayed higher elongations at break, perhaps as a result of the higher molecular weight of the base polymer selected as the backbone compared to PDMAEMA-Pent (250 kDa for the former compared to 68 kDa for the latter), and its reduced crosslinking density. The sharper increase in the apparent modulus observed for these samples is also in good agreement with the evolution of the shear moduli typically reported for these materials.^51^ This is presumably the result of more pronounced network defects generated at lower concentrations, associated with faster kinetics of cyclization and loop formation.^56,57^

However, interestingly, CMC-Allyl-based hybrids displayed adhesive failure profiles that contrasted with the cohesive failure observed for PDMAEMA-Pent-based samples (Figure S5). This was also associated with a more gradual fracture, resulting in seesaw profiles as hydrogels domains remained adhered to the elastomer surface and elongated prior to further fracture (Figure S4).

**Figure 4:**
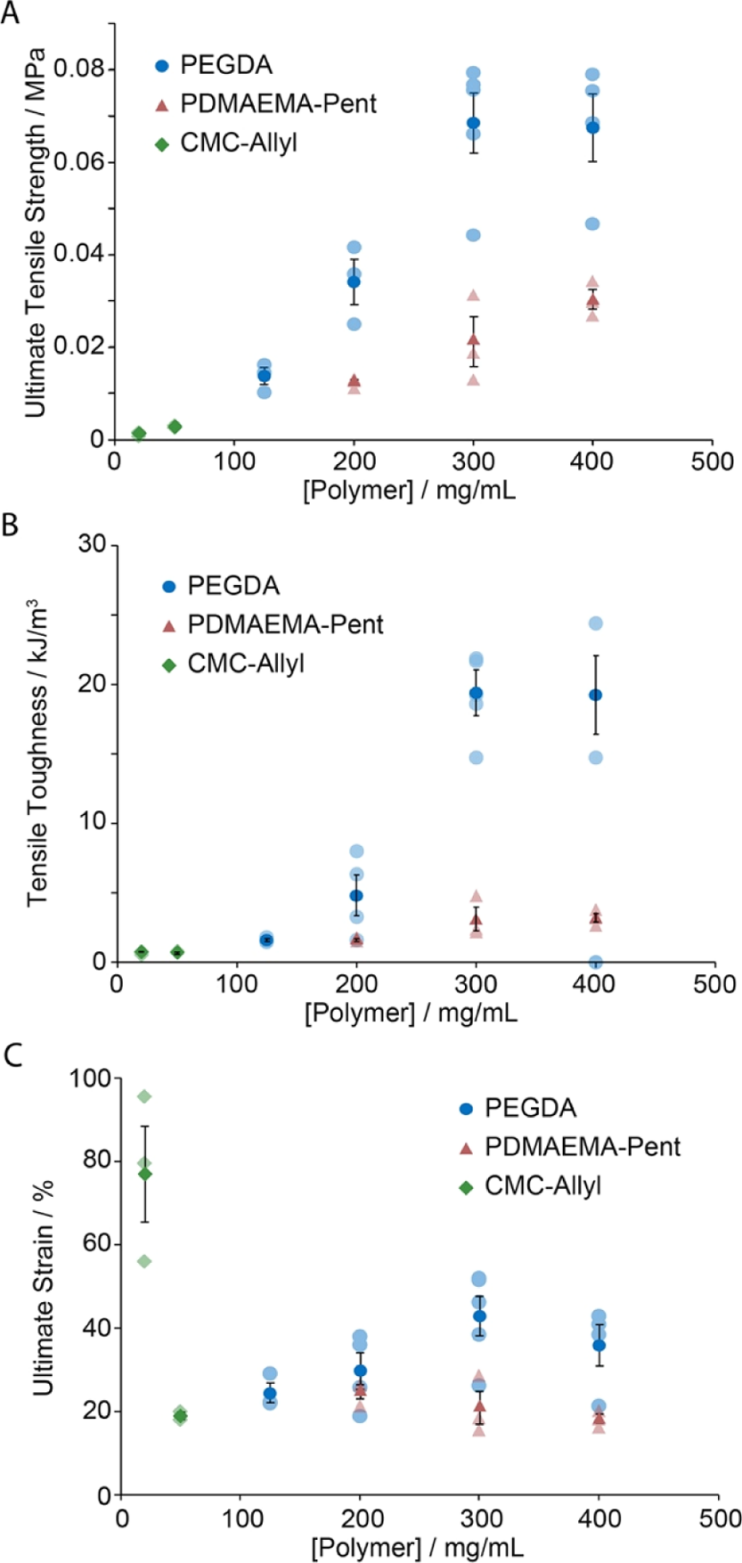
Summary of mechanical tensile properties of hybrid samples formulated with thiol–ene PDMS and different hydrogels [(A) ultimate tensile strength; (B) tensile toughness; and (C) ultimate strain at break]. Dark filled data points correspond to averages of ≥3 measurements ± standard errors. Light filled data points correspond to raw data points.

Therefore, the fracture profile and toughness of the hybrids were examined next (Figure 4). The ultimate tensile stress and tensile toughness of the hybrids generally increased as a function of polymer concentrations (Figure 4A,B). However, at the highest concentrations tested, the tensile toughness reached a plateau as a result of the lower ultimate tensile strains typically observed. This was particularly pronounced for CMC-Allyl hybrids, which displayed comparable tensile toughness at the two concentrations tested despite a clear increase in apparent modulus as a result of a sharp decrease of the ultimate tensile strain. This phenomenon is proposed to be associated with the adhesive failure observed in thiol–ene hydrogels, in particular at higher concentrations, as a result of the strengthening of the hydrogel phase.

**Figure 5:**
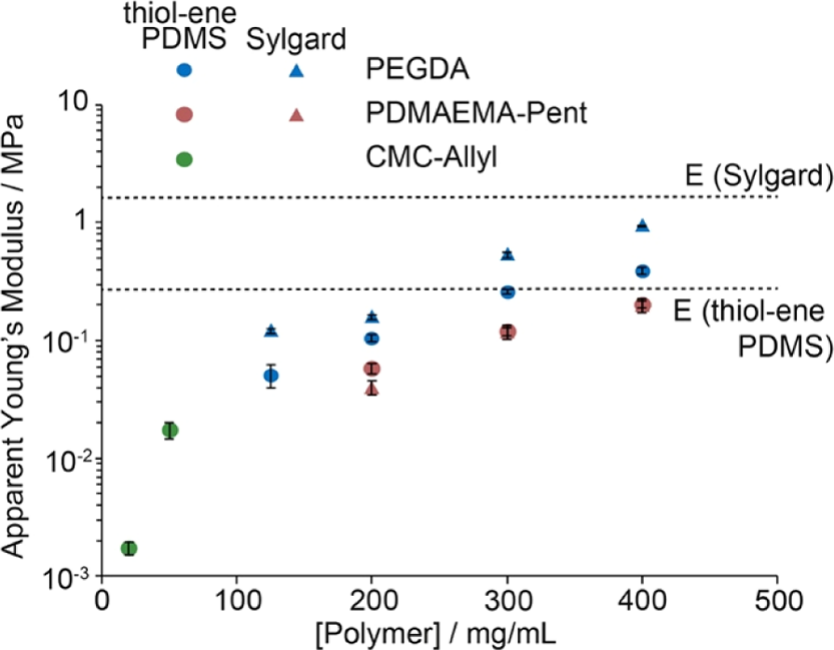
Comparison of the apparent Young’s modulus of hybrid samples formulated with thiol–ene PDMS or Sylgard PDMS (activated with PDMS-SH) and PEGDA or PDMAEMA-Pent hydrogels at different polymer concentrations.

The impact of the elastomer chemistry on the mechanics and bonding to hydrogels was investigated next. Hybrids were generated from Sylgard PDMS, displaying a comparable vinyl chemistry to thiol–ene silicones but crosslinking mechanisms relying on hydrosilylation. To promote bonding between the hydrogel network and the Sylgard silicone, a solution of PDMS thiol was applied to its surface prior to bonding. The impact of this change in elastomer chemistry on the apparent modulus of the corresponding hybrids was more pronounced in the case of PEGDA hydrogels compared to PDMAEMA thiol–ene hydrogels. Indeed, while apparent Young’s moduli were comparable in the case of hybrids formed with PDMAEMA thiol–ene hydrogels, when comparing hybrids generated with thiol–ene PDMS and Sylgard, the apparent Young’s moduli of PEGDA-based hybrids generated with Sylgard were systematically higher than those of the hybrids generated with thiol–ene PDMS (Figure 5). This different behavior could be partly explained by differences in bonding between the different phases in these hybrid systems, perhaps indicating improved coupling between PEGDA and the thiolated PDMS applied at the surface of Sylgard PDMS. Interfacial mechanics is not expected to have an impact on the apparent Young’s modulus significantly although it may the affect stress concentration at the interface. It is also possible that differences in the oxygen concentrations within Sylgard and thiol–ene PDMS differ, resulting in changes in crosslinking of the PEGDA phase. In contrast, the reduced sensitivity of thiol–ene coupling to oxygen concentrations results in a reduced impact on the apparent modulus of corresponding hybrids. Indeed, the oxygenation level of the hydrophobic substrate is known to have an impact on hydrogel mechanics, in particular close to the contact interface between the hydrogel precursor solution and the hydrophobic substrate.^58^ This would imply a higher oxygen content in thiol–ene PDMS compared to Sylgard, which we were not able to confirm experimentally.

**Figure 6:**
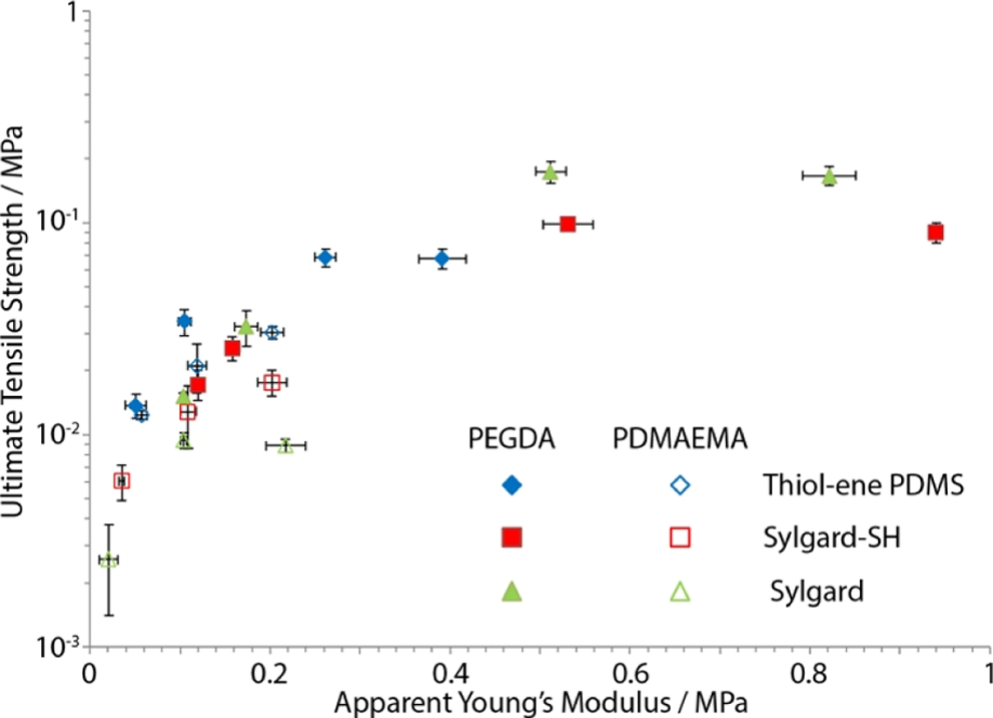
Comparison of the ultimate tensile strength of hybrid samples formulated with thiol–ene PDMS or Sylgard PDMS (with or without impregnation with PDMS-thiol, Sylgard, and Sylgard-SH) and PEGDA or PDMAEMA-Pent hydrogels at different polymer concentrations.

To explore the impact of the silicone elastomer chemistry on hydrogel bonding more directly, the fracture behavior and toughness of the corresponding hybrids were examined (Figure 6). In addition, to study the impact of thiol–ene bonding on hybrid mechanics and fracture further, we introduced another set of samples in which Sylgard PDMS was not coated with the solution of PDMS-SH. The PEGDA hybrids formed with higher concentrations of PEGDA all failed adhesively, possibly due to the overall relatively stiff and tougher character of the corresponding hydrogel phases, resulting in stress accumulation at the interface, dominating the fracture behavior. In agreement with this notion, comparison of the ultimate tensile strength of these hybrids indicated a plateau above an apparent Young’s modulus of 200 kPa (Figure 6). Therefore, these observations suggest that above such stiffness, the toughness of the gel phase and stress transfer concentrated at the interface, resulting in failure of the hybrids. In addition, in the plateau region, the ultimate tensile strength was higher in the case of the uncoated Sylgard hybrid compared to Sylgard hybrids with thiolated PDMS (Sylgard-SH) coating or thiol–ene PDMS. Therefore, at high hydrogel concentrations, the apparent Young’s modulus was sensitive to the surface chemistry of the Sylgard PDMS, perhaps reflecting the impact of the interfacial stress concentration on the overall deformation of the sample and suggesting that direct coupling of acrylates to Sylgard PDMS is more effective than coupling via thiol–ene bonding. In agreement with this notion, failure was systematically cohesive in these samples (Figures S6). At lower gel concentrations, the mechanical properties of the gel phase dominated the stress–strain profiles, and the sensitivity of the associated apparent Young’s moduli to the different silicones and interfaces explored for bonding was reduced. The bonding chemistry with the silicone had less impact on the ultimate tensile strength, perhaps reflecting that failure occurred more at the interface in Sylgard, both unmodified and treated with thiolated PDMS (Figure S6). Overall, PEGDA adhesion to Sylgard silicones appeared enhanced at higher hydrogel concentrations, based on the failure mechanism observed and the increased ultimate tensile strength observed compared to thiol–ene PDMS.

In the case of hybrids formed with PDMAEMA-Pent, the surface chemistry of the elastomer had little impact on the apparent modulus of the corresponding hybrids (Figures 5 and 6), in agreement with improved coupling between the two phases and potentially also the reduced sensitivity of thiol–ene chemistry to oxygen. The chemistry of the PDMS elastomer had a significant impact on the ultimate tensile strength of the corresponding hybrids, varying the strength of these materials by nearly 1 order of magnitude. As this phenomenon was not associated with a switch from cohesive to adhesive failure (Figure S6), the origin of this variation remains unclear. It could reflect a change in the stress transfer at the interface, differences in partial network interpenetration, or changes in the ultimate strain at break. Nevertheless, our data clearly indicate that bonding and toughness are enhanced when thiol–ene hydrogels are bonded to thiol–ene PDMS elastomers with a complementary imbalance of thiol moieties vs alkene residues (excess thiol in thiol–ene PDMS and excess alkene in the thiol–ene hydrogel).

**Figure 7:**
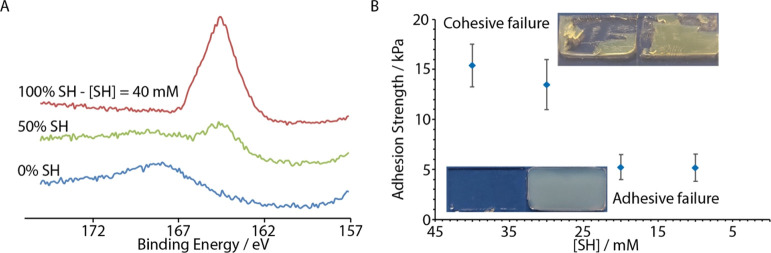
(A) S 1s XPS spectra of glass samples functionalized with mercaptopropyl trimethoxysilane at 0, 20, and 40 mM. (B) Adhesion strength extracted from shear lap testing experiments on PEGDA hydrogels (125 mg/mL) sandwiched between two glass slides functionalized with mercaptopropyl trimethoxysilane at different concentrations ([SH], indicated in mM).

Therefore, surface thiols promoted strong bonding of thiol–ene hydrogels, whereas conventional acrylate gels displayed reduced bonding. To further explore the impact of surface thiolation on the bonding of PEGDA hydrogels, a series of glass substrates functionalized with mercaptopropyl trimethoxy silane deposited at different concentrations were generated, and PEGDA gels were formed at their surface. Dilution of mercaptopropyl trimethoxy silane with trimethoxy(propyl) silane allowed us to control the density of thiol moieties (Figure 7A). Initial experiments explored the formation of hybrid structures comparable to the elastomer-based hybrids, in which cut glass slides sandwiched a gel phase side on. However, the resulting glass–gel–glass hybrids were too weak to enable tensile testing. This may partly be ascribed to the heavier weight of the glass substrates compared to the silicone elastomers, but it also suggests that a partial interpenetration of the hydrogel network within the elastomer network occurs at the interface, as is known to reinforce adhesion in other systems.^59−61^ Therefore, gels were sandwiched between two functionalized glass slides placed face to face.

The surface density of thiol moieties controlled by the solution concentration of mercaptopropyl trimethoxysilane had a strong impact on the adhesion strength and failure mode of the resulting samples (Figure 7B). At a low thiol concentration, samples failed at the interface and adhesion strength was low (near 5 kPa), whereas it increased to 15 kPa at higher thiol concentrations and resulted in the cohesive failure of the samples. The adhesion strength measured at higher thiol concentrations is comparable to the adhesion strength observed in the PEGDA-based hybrids with similar hydrogel concentrations. Therefore, the data obtained for PEGDA hybrids formed with three different types of silicones (thiol–ene PDMS and Sylgard with and without treatment with thiolated PDMS) indicate that coupling via thiols does occur, but it is less effective in strengthening the corresponding interfaces and ensuring cohesive failure than direct bonding via radical coupling between the acrylate network and vinyl residues of the silicone phase.

**Figure 8:**
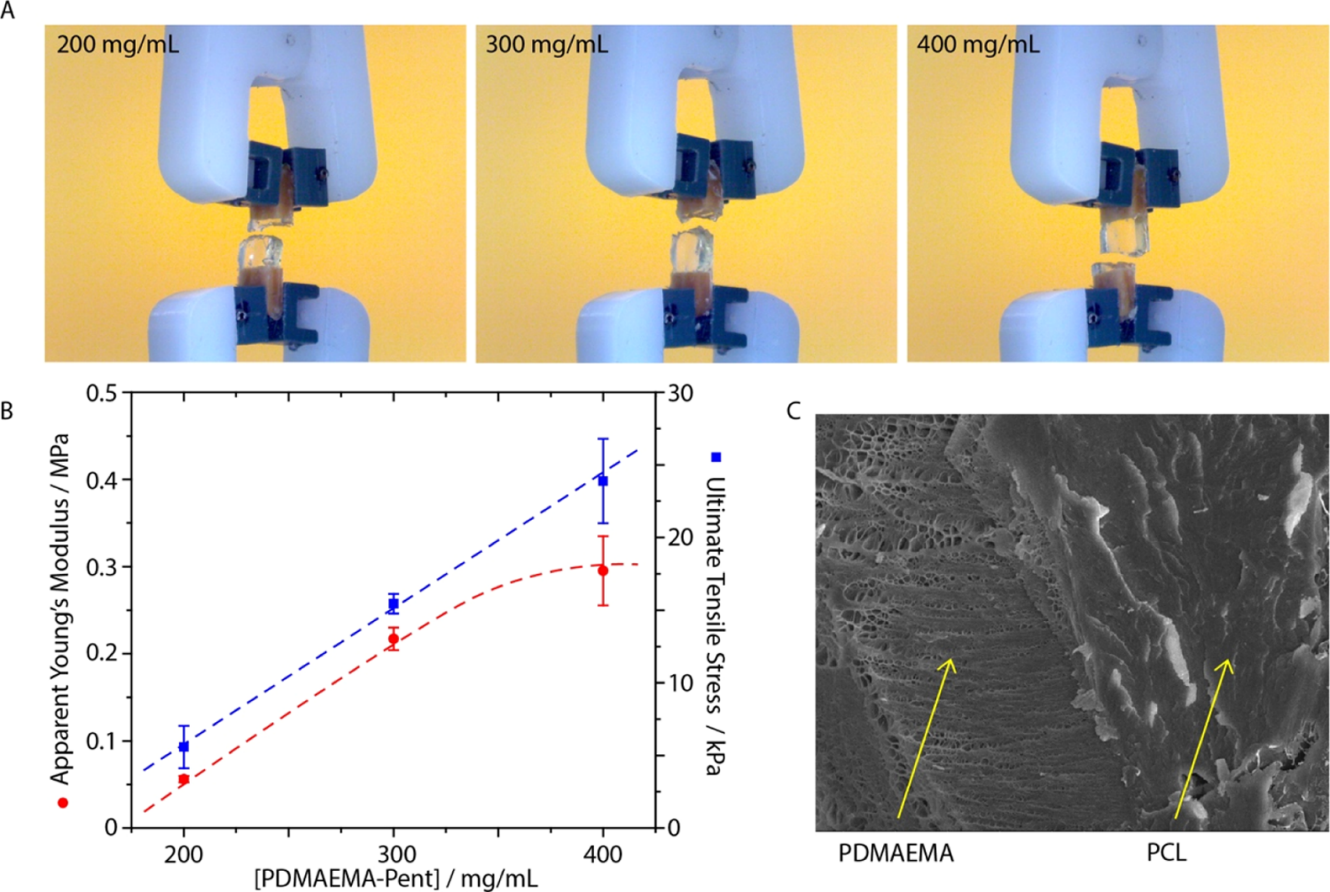
(A) Images of a hybrid sample (PDMAEMA-Pent hydrogel at different concentrations bound to two strips of propargylated PCL) stretched to failure. (B) Apparent Young’s modulus and ultimate tensile stress of corresponding samples. (C) Cryo-SEM image of the interface of a PDMAEMA-Pent/propargylated PCL after failure ([PDMAEMA-Pent] = 400 mg/mL).

Nevertheless, our data suggest that thiol–ene hydrogels can bond strongly to reactive elastomers and surfaces presenting alkene or thiol residues. This opens interesting opportunities to promote bonding with other cell instructive thiol–ene hydrogels, which are increasingly proposed as soft matrices for cell encapsulation and the regeneration of soft tissues,^1,2,10^ to more rigid polyesters that are typically applied to the regeneration of stiffer tissues such as bone.^44,46^ As a proof-of-concept, we explored the bonding of PCL samples to PDMAEMA-Pent hydrogels. PCL substituted with propargyl residues was synthesized by post-polymerization modification of PCL according to protocols previously published.^50^ The samples were then solvent cast into molds and cut and formulated into PDMAEMA-Pent-based hybrids. Within the range of polymer concentrations tested, failure was found to be cohesive (Figure 8A) and the ultimate tensile stress linearly increased, presumably reflecting the gradual increase in the gel modulus expected and apparent Young’s modulus measured (Figure 8B). Despite the excess alkene residues in the gel phase and the presence of alkynes alone in the PCL phase, the ultimate stress achieved remained comparable to that of hybrids formulated with the same gel composition and thiol–ene PDMS (compare Figure 8B to Figure 6). This is in agreement with the cohesive failure characteristic of both series of hybrids and indicates that strong bonding occurs between thiol–ene and thiol–yne networks, irrespective, to some level, of the position of the balance of moieties on both sides of the interface. To confirm the strength of thiol–ene bonding even with non-matched interfaces (alkyne excess in PCL and alkene excess in the gel), we imaged the interface of the corresponding hybrids after failure via cryo-SEM (Figure 8C). This clearly showed a well-integrated interface with no apparent crack, suggesting partial delamination. This is an interesting feature as it suggests that thiol–ene interfaces are relatively permissive in terms of the formulation of the gel and elastomer/hydrophobic phases, which should allow greater flexibility of the design of the corresponding materials and mechanical properties.

## Conclusions

Overall, the bonding of thiol–ene hydrogels to thiol–ene and thiol–yne elastomers and hydrophobic matrices appears particularly strong and seems to be limited by the surface density of the reactive residues in the absence of strong interpenetration of the two networks. Indeed, little infiltration of the gel network is expected into the PCL phase of propargylated PCL owing to its relatively high glass transition and crystallinity compared to silicones and melting temperature. Another interesting feature of the thiol–ene chemistry for the design of hybrid structures is the relative insensitivity of this chemistry to oxygen concentrations, therefore limiting variations in the apparent moduli that are observed in conventional radical polymerization-based hydrogels. Indeed, hydrophobic resins crosslinked via thiol–ene processes display little oxygen inhibition,^19,20,62^ and thiol–ene coupling in aqueous buffers was found to remain high even without degassing at moderately low thiol concentrations.^63^ The sensitivity of the ultimate tensile stress on the chemistry of the interface even in the absence of marked changes in the apparent Young’s modulus remains unclear, but we suggest that it could arise from changes in the ultimate strains at break observed, possibly reflecting slight changes in the network formation and associated energy dissipation or potentially highlighting the partial network interpenetration at the interfaces. In any case, our data clearly indicate that thiol–ene coupling at the interface of hybrid constructs offers great opportunities of design in corresponding multiphase elastomer–hydrogel structures generated from the microfabrication or 3D printing platforms. This could include the design of degradable polyester-hydrogel constructs with interwoven meshes in which degradable polyesters will play a load-bearing role or the engineering of microfluidic chips in which elastomers enable deformation of soft gel phases. In such systems, compliant, highly extensible gels, yet strongly bonded to elastomer interfaces, are predicted to enable the formation of soft tissues within associated phases without mechanical failure and delamination from hard elastomer structures. Considering the growing importance of thiol–ene elastomers and hydrogels in the engineering of microfluidic platforms and hard tissue engineering constructs and for the encapsulation of cells with cell-degradable hydrogels for soft tissue engineering, multi-phasic thiol–ene networks appear particularly well-suited for the rationale design predicted from a simple characterization of independently designed materials.

## Supporting Information Available

Images of hybrids during formation and after fracture in stress–strain experiments and additional stress–strain data (PDF)
