# Supplementary material for: Mechanical Evaluation of Hydrogel–Elastomer Interfaces Generated through Thiol–Ene Coupling
Source: ACS Appl Polym Mater. 2023 Jan 30;5(2):1364–73. doi: 10.1021/acsapm.2c01878 (PMC9926487; doi:10.1021/acsapm.2c01878)
Supplement: Supplementary file 1 — ap2c01878_si_001.pdf [file ap2c01878_si_001.pdf]

Supporting Information

Mechanical Evaluation of Hydrogel-Elastomer Interfaces Generated Through Thiol-Ene Coupling

Khai D. Q. Nguyen[1,2], Stéphane Dejean[3], Benjamin Nottelet[3], and Julien E. Gautrot[1,2]*

[1] Institute of Bioengineering and [2] School of Engineering and Materials Science, Queen Mary, University of London, Mile End Road, London, E1 4NS, UK.

[3] Polymers for Health and Biomaterials, IBMM, Univ Montpellier, CNRS, ENSCM, Montpellier, France

* *To whom correspondence should be addressed E-mail: j.gautrot@qmul.ac.uk.*

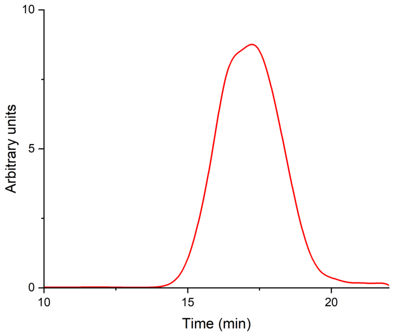

Supplementary Figure S1. Gel Permeation Chromatography trace obtained for PCL-alkyne.

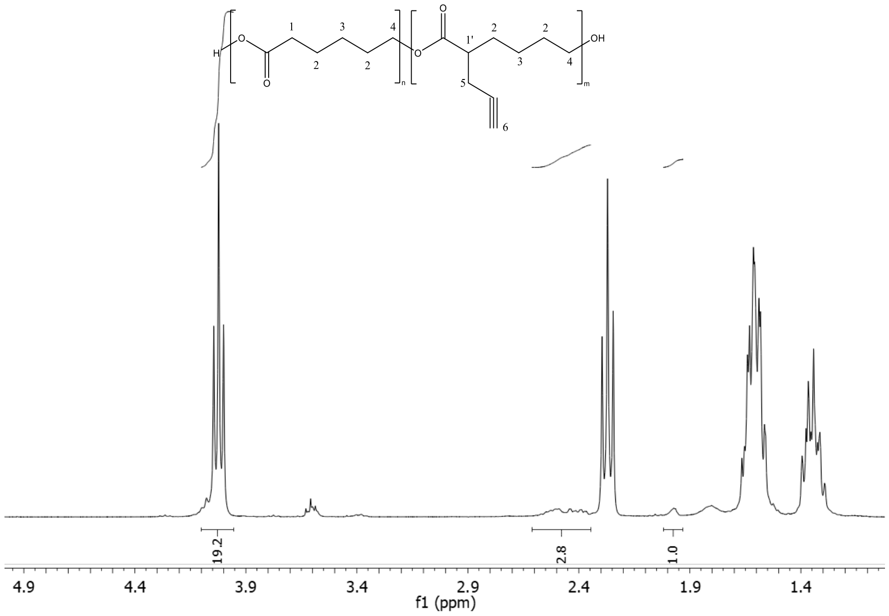

Supplementary Figure S2. ^{1}H NMR (300 MHz) spectrum of PCL-Alkyne ($CDCl_3$). Integrals required to calculate the substitution level are indicated.

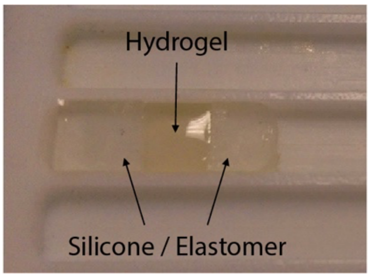

Supplementary Figure S3. Image of hybrid sample taken shortly after irradiation in a mould.

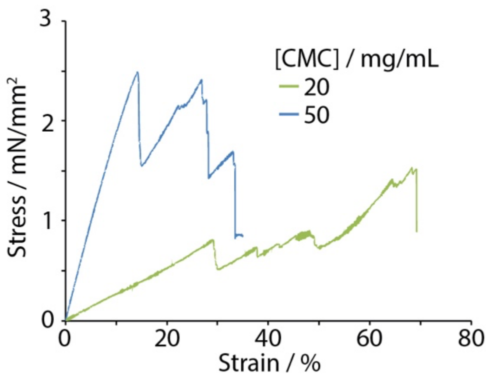

Supplementary Figure S4. Representative examples of stress-strain traces recorded for hybrid samples formulated with thiol-ene PDMS and CMC-Allyl hydrogels.

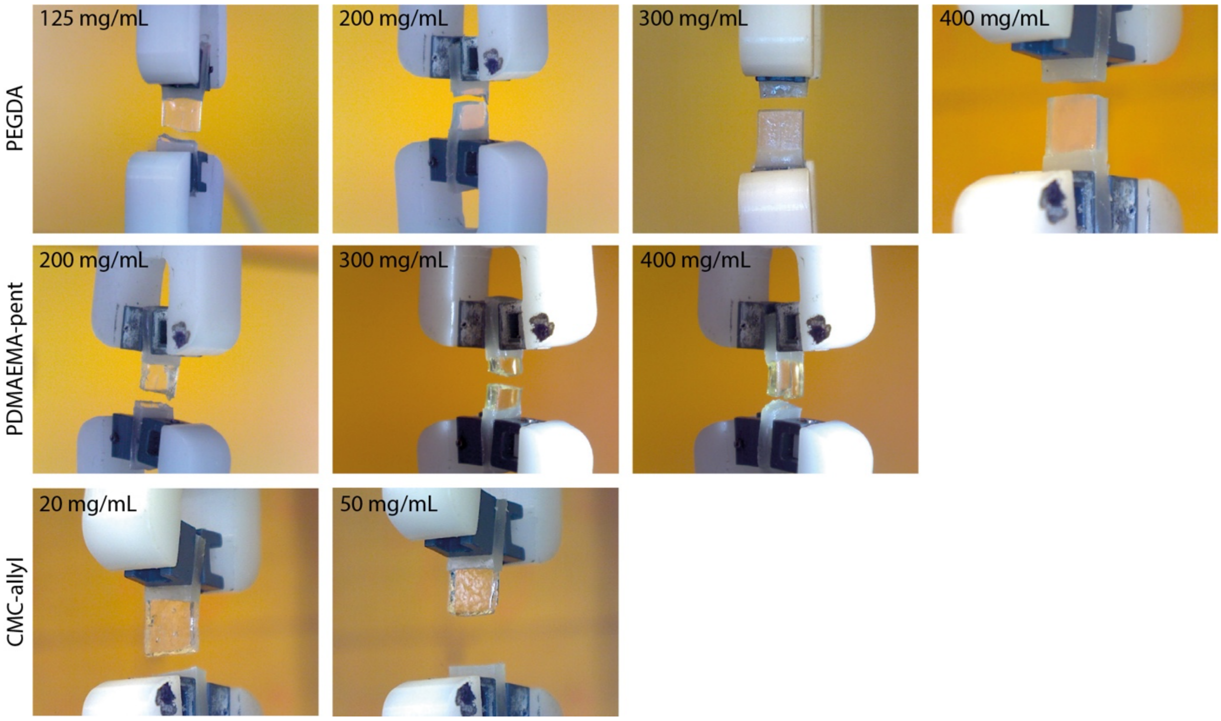

Supplementary Figure S5. Images of hybrid samples formulated with thiol-ene PDMS and various hydrogels, stretched to failure. The hydrogels used in corresponding samples was: A, PEGDA; B, PDMAEMA-Pent; C, CMC-Allyl.

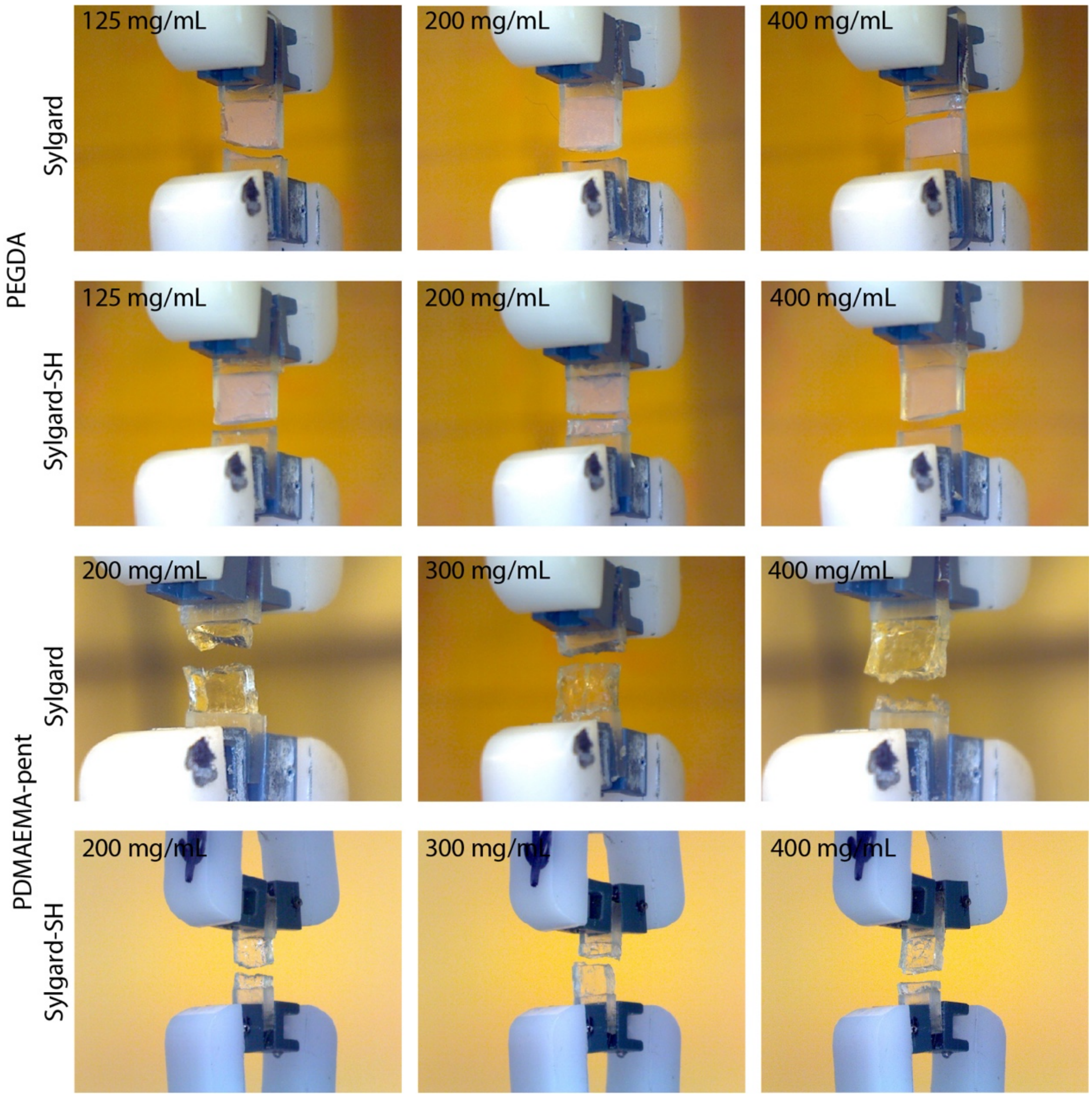

Supplementary Figure S6. Images of hybrid samples, after failure, formulated with PEGDA and PDMAEMA-Pent at different concentrations and Sylgard PDMS (with and without surface treatment with thiolated PDMS).
